# Final-note expectancy and humor: an empirical investigation

**DOI:** 10.1186/s40359-022-00936-z

**Published:** 2022-09-30

**Authors:** Sándor Imre Nagy, György Révész, László Séra, Szabolcs Ajtony Bandi, László Stachó

**Affiliations:** 1 Institute of Psychology, University of Pécs, Pécs, Hungary; 2 Brain Imaging Centre, Research Centre for Natural Sciences, Budapest, Hungary; 3 Faculty of Music and Visual Arts, University of Pécs, Pécs, Hungary; 4 Liszt Ferenc Academy of Music, Budapest, Hungary

**Keywords:** Music psychology, Psychology of humor, Empirical aesthetics, Melodic expectancy, Musical humor, Melodic incongruency, Final-note expectancy, Musical expectancy

## Abstract

**Background:**

Melodic expectations were manipulated to investigate the nature of tonally incongruent melodic final notes that may elicit humor in listeners. To our knowledge, this is the first experiment aiming at studying humor elicitation in music with the use of empirical, quantitative methods. To this aim, we have based the experiment on the incongruency/resolution theory of humor and the violations of expectations in music. Our goal was to determine the amount of change, that is, the degree of incongruency required to elicit humor.

**Methods:**

We composed two simple, 8-bar long melodies, and changed their final notes so that they could randomly finish on any semitone between an octave upwards and downwards with respect to the original, tonic final note. This resulted in 25 versions for both melodies, including the original final notes, for each semitone. Musician and non-musician participants rated each version of each melody on five 7-point bipolar scales according to goodness of fit, humor, beauty, playfulness, and pleasantness.

**Results and conclusions:**

Our results showed that even a single change of the final note can elicit humor. No strong connection was found between humor elicitation and the level of incongruency (i.e., the amount of violation of expectation). Instead, changes to the major-mode melody were more likely to be found humorous than those to the minor-mode melody, implying that a so-called playful context is necessary for humor elicitation as the major melody was labelled *playful* by the listeners. Furthermore, final notes below the original tonic end note were also found to be less humorous and less fitting to the melodic context than those above it.

**Supplementary Information:**

The online version contains supplementary material available at 10.1186/s40359-022-00936-z.

## Background

In the 1951 Merrie Melodies episode “Ballot Box Bunny” [1], [2], Yosemite Sam challenges Bugs Bunny to play the song “Those Endearing Young Charms” on the piano. Plotting against Bunny, Sam hides explosives in the piano, attached to the final note of the song. But much to Sam’s dismay, Bunny cannot find the correct note. In his frustration, Sam pushes Bunny away from the keyboard and plays the correct melody himself, triggering the explosives and eventually falling to his own trap.^1^

Since the publication of Leonard Meyer’s seminal book *Emotion and Meaning in Music* in 1956 [3]musical expectations – including the role of “hitting” the correct final note in melodies like in the above cartoon – have played a central role in music cognition research. Expectation is defined as a mental process that accounts for the prediction or anticipation of forthcoming events, based on previous experiences (for important overviews see, e.g., Eerola [4]; Huron [5]). As music unfolds over time as a sequence of sound events, it has implications [6], creating various levels of anticipation [5] or “yearning” [7] in the listener for the events in the sequence that are about to be heard. Emotions are induced by the suspension and/or the violation of expectations for a particular event or events, finally followed by a resolution.

According to Meyer [3]affect is aroused by the tension of inhibiting a tendency to respond to an expected event. Although Meyer was not the first to consider expectations as the basis of affect in music, his work inspired many theories such as those of Jones [8], Bharucha [9], Schmuckler [10], Narmour [6], [11], Margulis [12], Huron [5](for a review see [13], [14]. A large number of the experimental studies carried out in the subsequent decades examined expectancies of tonality [7], [10], [15], melody [4], [16–20], temporal structure [8], [21], and psychophysiological correlates of musical expectation [22–25].

## The study of musical expectations: research paradigms

Musical expectations have been studied using experimental paradigms aiming to identify when and how listeners form expectations of an incomplete musical stimulus, and what they expect the next element will be. Stimuli include pairs of tones [26], scales [15], sequences of tones or chords [27], [28], and cadences [29]. In some designs, participants are required to continue incomplete stimuli by singing [17], using a keyboard [28], or even continuing the incomplete stimulus in writing [30]. One drawback of these designs is that they require participants to be formally trained in music, to be able to imagine, sing, play, or write the notes they think are missing. Another is that participants can only provide one continuation, while in real-world music many continuations are possible, albeit to different degrees. One of the most popular methods of studying expectancy is the so-called probe-tone paradigm whereby participants rate how well a tone of random pitch (*probe tone*) fits or completes a scale, chord, or chord sequence that has already been presented [15]. This design provides insight into the strength of listeners’ expectations for particular tones at the end of a stimulus and shows the tonal stability of the probe tone in a given context [5], [31]. While this tonal stability establishes listeners’ “key profiles,” according to Krumansl and Kesssler [15], there is an ongoing debate as to the nature of the musical expectancies measured using this paradigm. For instance, Aarden [31] observes that because, in the original paradigm, the probe tone is presented in isolation, after the stimulus has been played, the results are more likely to represent listeners’ expectancies for phrase endings. Also, Arthur [32] claims that listeners’ higher ratings of tonic triad notes in a chord sequence are attributable not to tonal stability but the effects of the local chord context. Probe-tone paradigms thus reflect cadential expectations of melodies, addressed in tonal contexts (see [24], [29], [33] for cadential melodic expectations see [34], [35]). Our method in this study focuses exclusively on participants’ cadential melodic expectancies. Listeners’ note-to-note expectations change, as the music unfolds over time, according to what they have already heard [36]. Continuous rating methods have therefore been created to measure changing expectations. In a study by Nagel [37], for example, participants reported their emotional responses to music in real time by moving a pointer controlled by a mouse or joystick around a computer screen representing two-dimensional emotional space. Reaction time is also a valid measure in continuous rating experiments, based on the idea that, because an expected event is processed more rapidly, reaction time is shorter at the target event [5]. Participants are usually asked to listen to a priming chord or scale and then judge as quickly as possible whether the subsequent tone or chord belongs to the primed key [38], [39]. In other studies, listeners were asked to judge the progression of the melody contour after every melody note, in real time, while listening to the melody [31].

## Language, humor, and music

Music and language converge in many ways. Similarities between acoustic, structural, and syntactic processing, for example, have been revealed along with a significant neural overlap between the two domains (for some of the most important reviews see [40], [41], even to the extent that some theorists consider them to be different aspects of the same, broad communication system [42], [43].

Expectations also play a substantial role in empirical research on language processing. One sub-field of psycholinguistics focuses on the cognitive processes underlying the perception of humor, which is related to the violation of expectations. Raskin [44] claimed that, for a joke to be humorous, a necessary and sufficient condition is that it is capable, at least in part, of conveying information that can be interpreted in opposite ways. Listeners find a joke humorous when they realize that the information it conveys is ambiguous. According to Veatsch, “humor occurs when it seems that things are normal while at the same time something seems wrong” [45]. Incongruency-resolution (IR) theory became one of the most popular theories of humor in the second half of the 20th century (for a review see [46]). This theory is rooted in the arguments of Kant and Schopenhauer, among others, and was proposed by Suls [47]: the source of humor is incongruency, a paradox created by the collision of at least two contradictory elements in a conceptual framework. The occurrence of the second, incongruous, element results in a violation of expectation in cognitive processing. The incongruity is eventually resolved by a sudden insight that bears a similarity to the process of insight learning in Gestalt psychology [48]. Note, however, that incongruency and its resolution alone may not be sufficient for perceiving something as humorous. Wyer and Collins [49] extended the theory in their comprehension–elaboration model. In this model comprehension represents a combination of the phases of incongruency and its resolution, and elaboration represents a second phase leading to or overlapping with an affective appraisal of humor. Psychophysiological and brain imaging studies have corroborated this model by successfully distinguishing three stages in humor processing: incongruency detection, incongruency resolution, and elaboration [50], [51]. Influenced by studies of language processing, researchers have used the concept of incongruency, in the form of the violation of expectations, in many empirical studies of music (e.g., [22–24].

Even though humor in music is a common phenomenon and has been used extensively by composers in probably every era and genre (see[52]),^2^ it has only been addressed in a few studies to date. On the one hand, musical humor may relate to melody, tonality, musical structures, or external references, among others; on the other hand, it has also been associated with humorous stage performances and the lyrics of vocal music [53]. Some performers use humorous visual content to make the audience laugh, others use funny lyrics. In music(al) theater and opera, acting and characterization play important roles in eliciting humor. In most cases however, humorous music combines two or more elements. The comical characterization of operatic basso-buffo roles, for example, is achieved using a mixture of musical, acting, and verbal tools. This variability and diversity make humor in music difficult to study empirically. In our study we want to focus on humor elicited only by musical elements (i.e., humor in music without visual and lyrical cues)

In one of a very few early studies, Helen Mull [54]tested whether humor in music is coded inside the music itself or if it requires external cues such as the title of the piece. In more recent research, Sheinberg [55] and Bourne [56]studied irony as a form of humor in music. In his dissertation Plazak [57]measured the types of cues that performers use to express, and which listeners use to identify sarcasm in music, and then compared them with the cues used for identifying sarcasm in speech. Huron [53]observed and grouped expectation-violating musical “humor devices” (i.e., incongruencies, according to humor theory) in the pieces of P.D.Q. Bach, alias Peter Schickele. Huron categorized these as melodic (incongruous sounds), tonal (drifting tonality), metric (metric disruptions, implausible delays), stylistic (mixed genres, incongruous quotations), and structural (excessive repetitions). Rozin et al. [58] found that AAB patterns in music and verbal jokes produce the greatest humorous aesthetic effect. Randall and Moore [59] compared undergraduate students’ ratings of excerpts of humorous and non-humorous music and found a significant difference between the ratings of music majors and non-majors.

Morreall [60]suggests that listeners can have three different responses to incongruity: negative emotions, reality assimilation, and humorous amusement. Huron [5] states that a good indicator of “musical cultural understanding” is the comprehension of musical humor, in other words: the ability to successfully resolve incongruency. Huron [5] also proposes that three pivotal factors underlie the elicitation of laughter: the magnitude of expectation violation (i.e., incongruity), the recognition of a humorous intention, and, finally, the presence of a group, as people tend to laugh more often when they are in company. To our knowledge, no empirical study has yet been carried out to measure the relationship between the size of the incongruency in a melodic context and its humorous effect. In our study we focused on musical elements only, excluding visual and verbal cues^3^. Participants were asked to rate the humorousness of the melodies using one of five rating scales. To control for further possible latent variables, we reduced the number of incongruent elements by randomly changing only the final notes of two simple melodies composed by the first author. We also wanted to test whether this manipulation can result in perceiving the melody as humorous. The even distribution of semitones in an octave also provides the opportunity for gradual, scale-like responses, and for an empirically better characterization of the amount of incongruity required for humor perception.

Inspired by Huron’s proposals [5] the following questions emerged: (1) can a humorous effect be achieved by changing the pitch of the final note of a simple unaccompanied melody? (2) if so, do larger incongruous final notes result in higher humor ratings? (3) Are melodies in the major mode more or less humorous than melodies in the minor mode? (4) What effect, if any, does musical training have on the perception of humor in music? Our initial predictions, based on Huron [5], was that (1) even a single change in the melody can make it become humorous (2) and the more distant the final note from the tonic (and thus the greater the violation of expectations) the more the melody would be perceived as humorous. (3) If the degree of incongruity affects humor elicitation, then we expect that humor ratings would be consistent over major and minor modes. (4) And due to their more extensive knowledge about music, musicians will give higher and more consistent humor ratings.

## Methods

### Participants

Participants were 77 undergraduate and PhD psychology students (*n* = 55, 47 females), and current and former music students at the University of Pécs (*n* = 22, 12 females), whose ages ranged from 18 to 55 (*M =* 22.49, *SD =* 5.93). All reported normal hearing and normal or corrected to normal vision and no history of neurological disorders. Further, all were familiar with using a desktop computer and a mouse. The language of the experiment was Hungarian, and all participants were Hungarian native speakers.^4^

### Materials

**Fig. 1:**
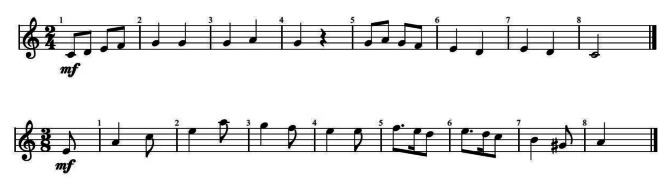
The two melodies in C major and A minor

The stimuli were two simple, four-measure monophonic melodies composed by the first author, one in C major with a 2/4 time signature and the other in A minor with a 3/8 time signature (Fig. 1). They both contained 18 notes and lasted for 8 s (major: 120 bpm, minor: 90 bpm). The harmonic minor scale was used in the A-minor melody with the presence of G# as a leading tone preceding the final note, strengthening tonal expectations.

We composed the melodies in such a way that they would conform to Western listeners’ heuristic expectancies of melodic organization (cf. [5]). Both stimuli had a clear arch-shaped pitch contour; also, they mostly used small intervals, thus creating a central pitch tendency, and conforming to pitch proximity. The second part of both melodies descended mostly in small steps, thus meeting the common statistical tendency of step declination. The minor melody started with the dominant on the upbeat and the tonic on the downbeat of the first measure before ascending in leaps no greater than a perfect fourth. Both melodies ended on the tonic. To avoid boredom and thus negative humor evaluations, the two melodies differed in several respects (including rhythmic and intervallic-step differences), but as mentioned above, both were fully conforming to Western listeners heuristic melodic expectancies.

We were also interested in finding out whether there was any effect on participants’ ratings of the final note being higher or lower than the tonic) as, to our knowledge, this had not been addressed in previous research.

Each melody was created with the software GuitarPro, version 6 (Arobas Music, France), using an Acoustic Piano soundbank with the Realistic Sound Engine turned on (Microsoft GS Wavetable Synth, Redmond, WA), and then exported as a .wav file at a sampling rate of 44.1 kHz and 16-bit amplitude resolution. We used SilverLine HS-55 V headphones, pre-tested by each participant before the experiment; also, each participant adjusted the volume to the level that suited them. The experiment was created and presented to the participants using Opensesame, an open-source, Python-based software [61].

### Design and procedure

At the beginning of the experiment, we asked participants to provide the following data: gender, age, and musical training (i.e., how long they had been playing an instrument, singing in a choir, or attending solfeggio [aural skills] classes). They were asked to round down the year values if these were not whole numbers; thus, for example, less than one year of study was represented as 0. Participants who had at least 10 years of experience in any of the musical activities mentioned (instrument, choir, or solfeggio) were assigned to the group of musicians, while all others were classified as non-musicians. We presented participants with the two original stimuli, which they rated using five 7-point bipolar rating scales. The two extremes of the scales were labeled as antagonistic qualities representing (1) how well the final note fitted the melody (with the closest English translation of the original terms in Hungarian (*Did not fit at all – Absolutely fit into it*) and (2) how do you feel about the melody, with four sub-scales *Not humorous – Humorous/Funny*, (3) *Serious – Playful*, (4) *Irritating – Pleasant*, and (5) *Ugly –Beautiful .* (For the original terms in Hungarian, see the Supplementary material.)

To avoid any potential bias in the ratings, participants were told that they were taking part in an experiment in musical aesthetics and were only informed about the ultimate purpose of the study after the experiment. The two variables of primary interest were those represented by the first and fourth rating scales, goodness of fit and humor; the others were distractors included only to avoid rating bias. To gain a better insight into the complexity of aesthetic responses, we decided to include the distractor variables in the statistical analyses.

In the practice phase of the experiment, we presented the two stimuli shown in Fig. 1, and the rating scales, to familiarize participants with the melodies and make it easier for them to rate them by creating strong final-note expectancies. In the experimental phase, we presented the stimuli with different final notes, having told participants only that the final note of each melody would be changed and that the changes were random. The melodies could end on any semitone ranging from an octave below to an octave above the final note of the original stimulus (i.e., the tonic): from C_3_ to C_5_ for the major melody and from A_3_ to A_5_ for the minor melody. The two stimuli were presented in randomized order so that participants could not guess whether they were about to hear a minor or a major melody. They heard all 25 possible endings for each of the two melodies and were asked to rate the whole of each melody including the final note using each of the five rating scales as soon as they had heard it. All participants confirmed that they clearly understood the instructions, and the duration of the experiment varied between 25 and 40 min.

### Statistical analyses

We used a Microsoft 365 Excel spreadsheet (Version 2009 build: 13231.20390) to create the database and calculate mean ratings; we used the free, open-source R-based software, JASP [62] to carry out the statistical analyses and create figures and tables.

## Results

### Descriptive statistics

**Fig. 2:**
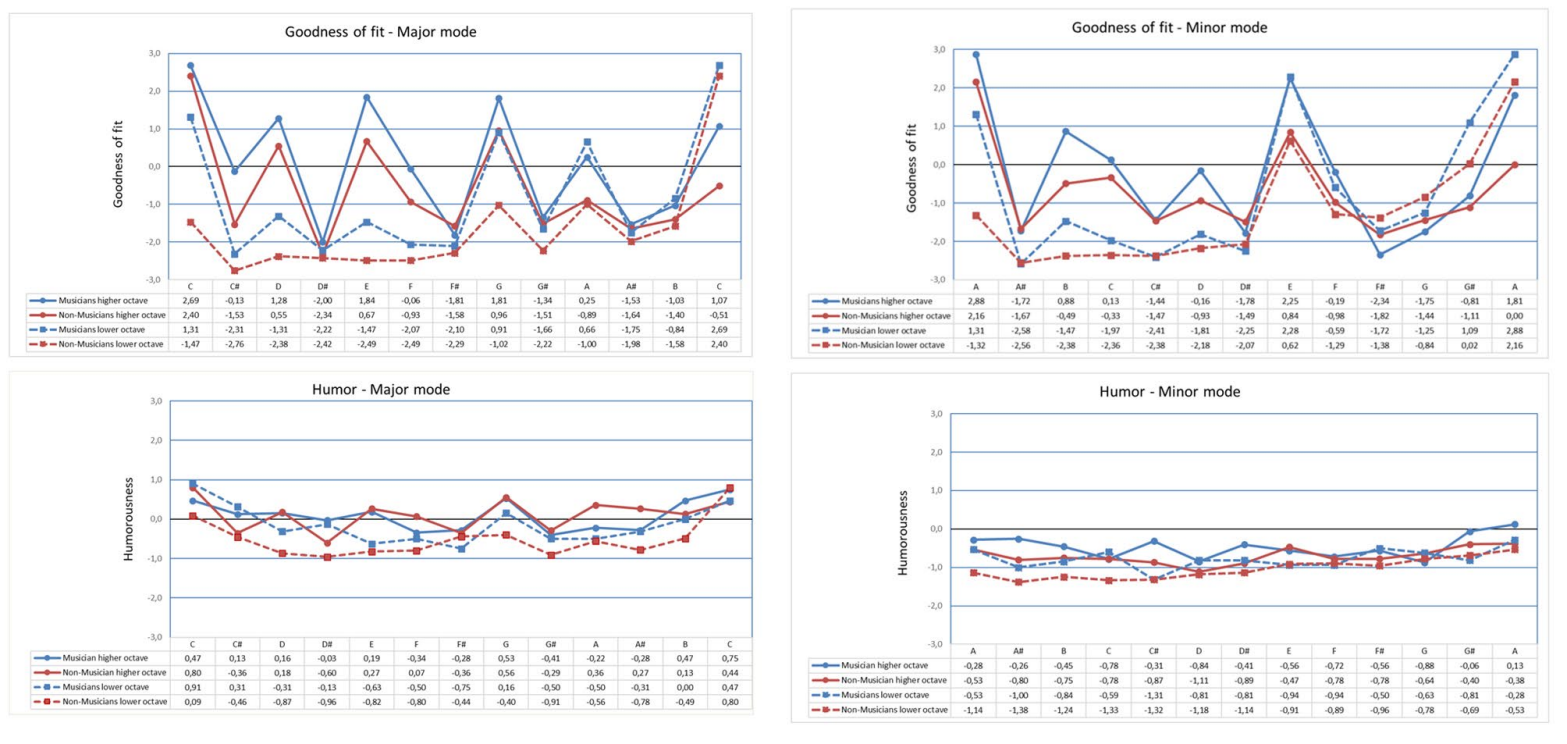
Means of goodness of fit and humorousness ratings in major mode. b Means of goodness of fit and humorousness ratings in minor mode Note. The bipolar scales were presented as antagonist pairs of qualities ranging from the value -3 to +3 (e.g. Not humorous ?Humorous; Did not fit at all ? Absolutely fit into the melody). The x axis represents the final notes with the lower and higher octaves folded, so that each pitch class is arranged vertically. Because of this, it is important to note that the higher tonics of the lower octaves are the same notes (and values) as the lower tonics of the higher octaves

We calculated the means of participants’ ratings of each of the major and minor melodies for each aesthetic category. Ratings of the goodness and fit and humor of the major and minor melodies are shown in Fig. 2. Negative values represent the negative side of the antagonistic pairs (e.g., *non-humorous or non-fitting*), 0 values represent neutral responses and positive values represent the positive side (e.g., *humorous or better fitting*). The higher the value, therefore, the more humorous the melody or more fitting the final note was rated. The x axis represents the final notes, the lower and higher octaves folded on to the axis, so that each pitch class from different octaves are arranged vertically with their respective mean values below the final notes. A total of 500 means was obtained: 2 (musicians vs. non-musicians) x 2 (major and minor melodies) x 5 (rating scales) x 25 (final notes).

Mann–Whitney U tests revealed a significant difference between musicians’ and non-musicians’ median ratings of humor for the minor melodies (*Mdn* = − 0.63 and − 0.87 respectively), U = 183.5, *p* = .006) but no difference between musicians’ and non-musicians’ median ratings for the major melodies (*Mdn* = − 0.13 and − 0.36 respectively), U = 239.0, *p* = .078. Both groups rated major melodies more humorous than minor melodies, U = 542.0 (musicians) and U = 531.5 (non-musicians), both *p* < .001.

Musicians rated minor melodies ending on a note higher than the tonic significantly more humorous (*Mdn* = − 0.51) than those ending on a lower note (*Mdn* = − 0.83, U = 30.5, *p* < .009), but the difference between their ratings of major melodies ending on higher and lower notes was non-significant. Non-musicians also rated minor melodies ending on a higher note more humorous (*Mdn* = − 0.78) than those ending on a lower note (*Mdn* = − 1.14, U = 17, *p* < .001), and major melodies ending on a higher note more humorous (*Mdn* = 0.16) than those ending on a lower note (Mdn = − 0.67, U = 10, *p* < .001).

Non-musicians rated major melodies ending on a higher note higher for goodness of fit (*Mdn* = − 1.17) than those ending on a lower note (*Mdn* = − 2.26, U = 22.5, *p* = .002) but there were no significant differences between the goodness-of-fit ratings assigned by musicians or non-musicians to minor melodies ending on higher and lower notes.

(1) We predicted that even a single change in the melody can make it sound humorous, and as shown in Fig. 2, positive humor ratings in the major mode confirmed our predictions. However, in minor mode (Fig. 2) there were almost no positive values in humor ratings indicating that other factors might also contribute to humorous appraisal. (2) Our second prediction was that the more distant the final note from the tonic (thus the greater the violation of expectations and the larger the incongruity), the more the melody would be perceived as humorous. This prediction was not confirmed: humor ratings were higher for the more congruent final notes, especially for the tonic and the dominant, and tended to be lower for the more incongruent ones. (3) We also predicted that humor ratings will be consistent over the two modes, but as mentioned above there was a significant difference, indicating a divergence in humor appraisal between major and minor modes. (4) Finally, we predicted that due to their more extent experience with music, musicians tend to have higher humor ratings. This prediction was also disconfirmed because, we only found a significant difference in minor mode ratings. An interesting addition was that both musicians and non-musicians in both modes found higher octave changes more fitting and more humorous.

### Correlations

As shown in Table 1 we calculated correlations between the mean ratings of each of the melodies for each of the five aesthetic qualities (goodness of fit, humor, playfulness, pleasantness and beauty). All correlations were significant (*p* < .001 or *p* < .002 in the case of the correlation between playfulness and beauty). Contrary to our prediction, 2) goodness of fit only moderately correlated with humor ratings (*r*_*s*_(98) = 0.438), but humor strongly correlated with playfulness (*r*_*s*_(98) = 0.893). Goodness of fit was very strongly correlated with both pleasantness and beauty (*r*_*s*_(98) = 0.930 and 0.933 respectively) while pleasantness and beauty were so strongly correlated (*r*_*s*_(98) = 0.980) We can infer these terms to have similar meanings for listeners. In future research they could be replaced by a single term, or one of them could be omitted. There was also a strong correlation between humor and playfulness (*r*_*s*_(98) = 0.89).

**Table 1:** Correlation matrix of the aesthetic qualities

Variable	goodness of fit	humor	playfulness	pleasantness
goodness of fit	—			
humor	0.438 ***	—		
playfulness	0.352 ***	0.893 ***	—	
pleasantness	0.930 ***	0.377 ***	0.327 ***	—
beauty	0.933 ***	0.352 ***	0.311 **	0.980 ***

*Note.* Spearman’s rho correlation. * p < .05, ** p < .01, *** p < .001

### Network analysis

**Fig. 3:**
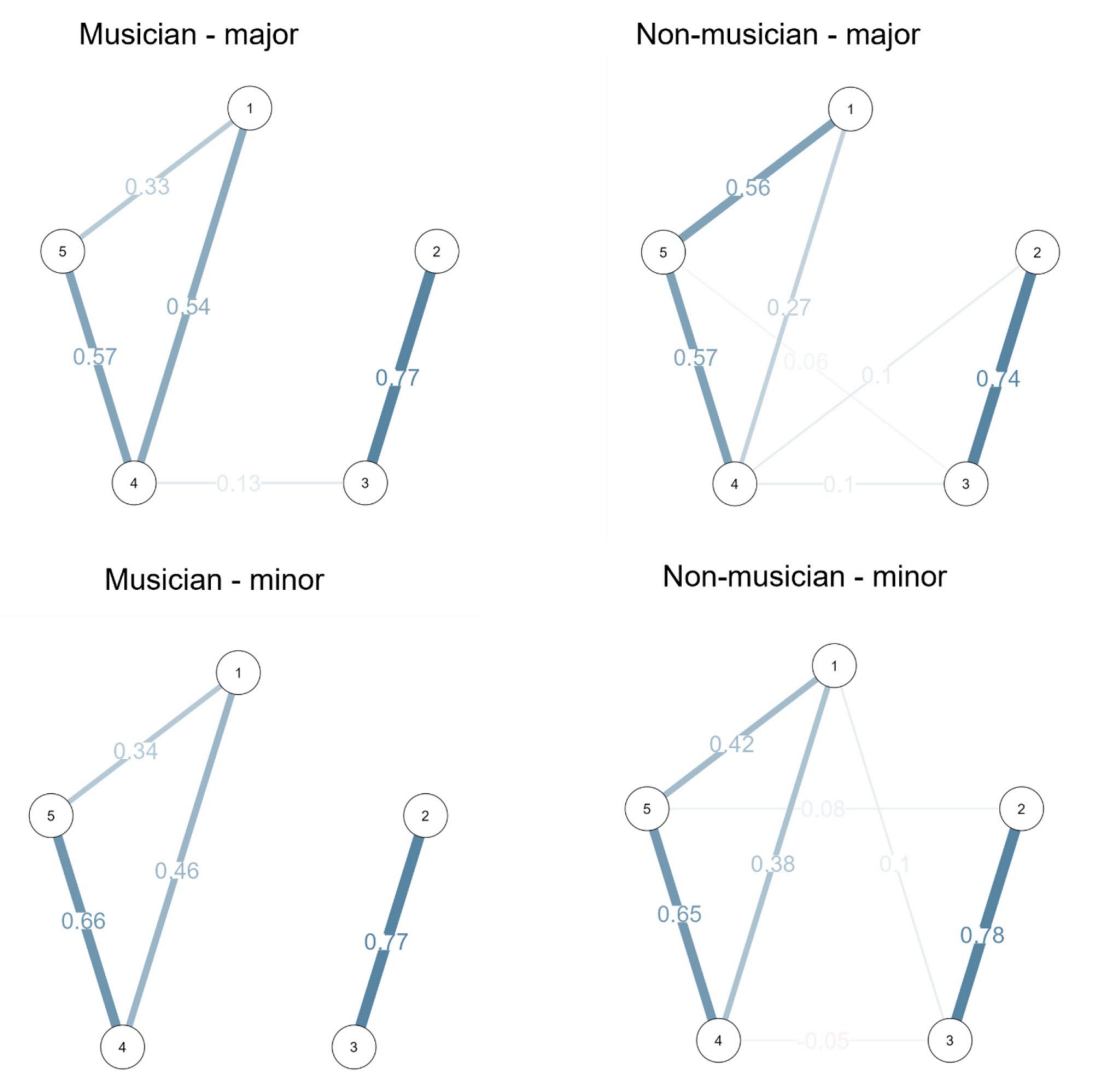
Network analysis *Note*. 1 = goodness of fit; 2 = humor; 3 = playfulness; 4 = pleasantness; 5 = beauty

We conducted a network analysis of ratings of all the melodies for each of the five aesthetic variables separately for each mode (major and minor) and for musicians and non-musicians, applying EBICglasso estimation, normalized centrality measures, and the tuning parameter (λ) set to 0.5. Visual inspection revealed two distinct groups, the first including goodness of fit, pleasantness, and beauty, and the second including humor and playfulness. We observed strong connections between the variables in the two groups and weaker connections between goodness of fit and humor (Fig. 3). (For the detailed network summary and centrality plots see the Supplementary material).

### Cluster analysis

To explore how participants’ ratings of goodness of fit, playfulness, and humorousness grouped together for each of the 25 major and 25 minor melodies, we conducted a cluster analysis (see Supplementary material for the details, including a color-coded table) and, to examine the results more closely, a series of separate cluster analyses for each mode and group (non-musician–major, musician–major, non-musician–minor, minor–musician). The parameters and the method were the same in each case. The Fuzzy C-means clustering method in JASP was used, similarity measures were based on the Euclidean distance, and the number of clusters was determined using the Elbow method and optimized by the Bayesian Information Criterion (BIC). The algorithm’s fuzziness parameter was set to 2 in each case and the maximum number of iterations was 25.

We labeled the resulting clusters based on their cluster means of the above-mentioned three categories. For the detailed cluster means, centroids, and t-SNE plot see the Supplementary material; we report here only the least-extreme mean value of each of the categories analyzed: goodness of fit, humor, and playfulness. For major melodies, we found two clusters for both musicians and non-musicians: a high-appraisal group (least-extreme cluster mean for musicians > 1.1, non-musicians > 0.9), and a low-appraisal group (both musicians and non-musicians < -0.53). In minor melodies, the algorithm grouped the data into three different groups. Based on the cluster means of the analyzed categories, we labeled these groups as we had for major melodies: high appraisal (musicians > 0.9, non-musicians > 1.5) and low appraisal (musicians: < -0.1, non-musicians < -0.9), and we labeled a third group of musicians as mixed appraisal, with low ratings of goodness-of-fit and high ratings of humor and playfulness. Finally, we labeled a third group of non-musicians as moderate, with cluster means varying between 0 and 0.25.

## Discussion

In the present study we predicted that (1) a humorous effect can be achieved by changing only the pitch of the final note of a simple unaccompanied melody. (2) and the more distant the final note from the tonic, and thus the greater the violation of expectations, the more the melody would be perceived as humorous. Our first prediction was partially confirmed, even a single change can result in positive humor appraisal, but only in the major mode. However, there was no clear relationship between the degree of incongruency and humorousness ratings, and contrary to our prediction those final notes tended to receive higher humor ratings which also had higher goodness of fit ratings. This was also supported by the moderate correlation between goodness of fit and humor (ρ = 0.438, p < .001). Our prediction (3) that humor ratings will be similar in both the major and minor melodies, was also disconfirmed: major melodies received significantly higher ratings. And finally, (4) we predicted that musicians’ and non-musicians’ ratings would be different, but our results showed that the ratings had similar tendencies. We also found that musicians and non-musicians in both modes gave higher humor ratings for those final notes that ended higher than the original tonic.

### Goodness of fit ratings

Goodness-of-fit ratings replicated Krumhansl and Kessler’s [15] findings. In their study, participants’ goodness-of-fit ratings were defined as a hierarchical organization of key profiles, i.e., a “tonal hierarchy”. According to Aarden [31], such ratings represent listeners’ schematic expectancies of melodic closure which are different in major and minor modes. As it was expected, in the major mode, our listeners’ goodness-of-fit ratings of the final melody note were highest (in the following order of magnitude): for the tonic (including the original, one octave up, and one octave down, that is: C_4_, C_3_, C_5_), then the dominant ending (for musicians, both upwards and downwards: G_4_, G_3_), the mediant (E_4_), the supertonic (D_4_), and the submediant final notes (for musicians, both upwards and downwards: A_3_, A_4_). This latter might be the result of interpreting each of these endings as a deceptive cadence, which is a common ending type in Western classical music and reflects musicians’ more extensive familiarity with these phrase endings. In the minor mode, the following final notes received the highest goodness-of-fit ratings (in order of magnitude): the tonic (the original, and both upwards and downwards: A_4,_ A_5_, A_3_), the dominant (both upwards and downwards: E_5_, E_4_), the supertonic (B_4_), and the leading-note ending (G#_4_) (this latter two for musicians only; cf. Figure 2a and b). In general, musicians gave slightly higher ratings for almost every possible ending, but it is to note that the difference was significant only in the minor mode. Although scale degrees of the root triads stood out with higher rates, interestingly, in contrast to Krumhansl and Kessler’s [15]early findings, our results showed a lower preference for the subdominant (F_4_ in major and D_5_ in minor) as final melody note. Similarly, we could not observe positive ratings in the minor mode for the mediant (C_5_) and the submediant (F_5_). It is possible that these differences can be accounted for by the shortness of the melody which enables the listener to reinterpret it as a half-period, making it possible to activate different schematic expectations than in Krumhansl and Kessler’s [15]study, where stimuli of various lengths were used.

While conducting the analysis we were also interested in learning whether the direction of the final interval had any effect on participants’ ratings. Participants found that final notes finishing higher than the original were more fitting (although this effect was not significant in the minor mode, probably because the minor mode had generally lower goodness-of-fit ratings). A potential explanation to this is that the contours of the actual melodies never dipped lower than both the starting and the “correct” (most expected) final tonic note, thus forming weaker expectations about intervals that finished lower than the lowest note of the melody contour.

### Humor ratings

In general, only a relatively small number of humor ratings were positive (that is, closer to the humorous end of the scale); also, these positive ratings were relatively low. In the major mode, musicians more often interpreted final-note changes as humorous, and with higher intensity, although the effect was not significant. In the minor mode, however, the difference was significant; but except for musicians’ minimal positive Humor rating of the upper octave (A_5_), all ratings were at the negative, “non-humorous” side of the scale. In keeping with the goodness-of-fit ratings, final notes ending lower than the correct, most expected tonic final note were found by all participants even less humorous than steps ending higher than the tonic final note in both modes (Fig. 2). It is important to note, that Arthur [32]found that musicians and non-musicians were fairly consistent in their responses when asked to rate the evoked qualia in a probe-tone paradigm. In contrast, as already mentioned in the introduction, Randall and Moore [59] found that music majors had significantly higher preference and perception for humor than non-majors.

It is also important to highlight that it was the most expected note (the tonic) that consistently received the highest ratings in major mode from both musicians and non-musicians (see Fig. 2). Among the final notes, the few exceptions that made the melody sound humorous for the participants without high goodness-of-fit ratings were the lower supertonic (C#_3_) and the leading note (B_4_) for musicians in the major mode – that is, the only non-diatonic final notes. A possible explanation for these exceptions relates to the fact that due to their more extensive experience, musicians might have been able to reinterpret these steps as transient notes moving towards the tonic. Incongruency (error) detection is a necessary but not sufficient condition of perceiving humor: a *resolution* is also required that leads to positive affective evaluation. As already mentioned in the introduction, to successfully resolve the incongruency in a given context in order to elicit humorous aesthetic emotional responses, one needs to be familiar with the contextual framework in question – that is, s/he needs to be able to form stable cognitive representations in order to create a proper humorous response [63], thus as Huron states, musical humor perception is an “acid test for musical cultural understanding” [5]).

### Humor and playfulness

Further analyses revealed a significantly high correlation between playfulness and humor. The high correlation between playfulness and humor might suggest why there were no positive humor ratings in the minor mode: the minor melody received significantly lower playfulness ratings (Mdn = -0.41) than the major melody (Mdn = 0.18) (U = 2259.5, p < .001), and this has probably undermined the humorous evaluation of the various end notes of the minor melody. In fact, in line with our findings, listeners associate minor mode with sadness, sorrow, darkness, melancholy, etc., which contrasts with happiness, playfulness, and humor (see Hevner [36]). Also, the author of this classic study [36] put the adjectives “playful” and “humorous” in the same group among the fourteen adjective groups used in her study from which participants had to choose those what seemed the most appropriate to the heard music. The difference between major and minor modes was also significant regarding listeners’ votes of humor and playfulness, similarly to our findings: overall, they found the major melodies more humorous and playful than the minor melodies. Half-a-century later, Asmus [64] developed a questionnaire to assess emotion and affective states in responses to music. One of the 9 dimensions he created through a principal component analysis of originally 99 adjectives was labeled as “humor”. In this study, the categories “humorous” and “playful” were loaded onto this dimension.

Furthermore, the convergence of playfulness and humor ratings might have been due to the fact that participants likely have no fixed representation of humor as a concept (and probably due to fatigue effects the subjective sensation of humor is also changing over time); and without such a clear distinction, the two concepts tend to merge into one subjective notion. An alternative explanation is that participants might have routinely interpreted the two qualities as synonyms.

We also have to mention, that humor theorists pointed out that an important factor of a successful and eventually pleasurable resolution of a perceived incongruency is that the situation, or context, in which it occurs must be “safe” or non-threatening [47], [65], [66]. One can hardly imagine any non-safe context in a musical melody, but the underlying cognitive and affective mechanisms that can positively evaluate incongruency as humorous are most probably similar. In other words, perceiving something as humorous requires a specific cheerful or playful affective state of mind (cf. [46]). In our case, the most probable explanation is that the simplicity and childlike tuneof the major melody has activated this “playful mode”, resulting in the humorous appraisal of the changes in the final tone.

A cluster analysis including the variables of goodness of fit, humor, and playfulness further corroborated our findings (see Supplementary material). In the major mode, those final notes were found to be more humorous that fitted better the melody line; and based on the cluster means, we found no cluster that would convincingly provide evidence for non-fitting but humorous ratings. A few final-note ratings, however, differed between groups. Some ratings by non-musicians fell into the high-appraisal group for the end notes F, A, A#, and B, but ratings for these notes were very close to cluster boundaries (showing no clear distinction from the low-appraisal cluster); and another cluster analysis (with different parameters) might have classified these ratings differently (for the t-SNE cluster plots see the Supplementary material).

Regarding the direction of the final step, the difference between musicians and non-musicians was clear: non-musicians found downward steps less fitting and humorous than musicians, likely indicating that these latter might have been able to successfully resolve incongruency on a broader cognitive framework (cf.[46]). In the minor mode, the results were more mixed, arguably because of the generally low values of humor and playfulness ratings. Among musicians, we found a third cluster, called “Mixed”, which contained non-fitting but humorous and playful final notes. These steps include more non-diatonic steps, but in this cluster, the low values of humor (mean = 0.49) and playfulness (mean = 0.56) do not indicate convincing evidence of incongruency–humor coupling. The analysis also revealed a third cluster (“Moderate”) in non-musicians’ minor ratings. In this cluster, average values converged around 0 (neutral ratings) and fell between the “High” and “Low” clusters. Interestingly, in non-musicians, the lower leading tone (G#) was classified into the “High” cluster.

## Limitations and further research

A reductionist approach to a complex phenomenon such as humor naturally has its limitations. If we aim at studying the components of humor processing separately, there is a high risk that we lose humor itself, especially if the stimuli are oversimplified. In our exploratory study, we aimed to create melodies, and modified versions thereof, that are (1) simple enough to be able to study the role of melodic expectancy in creating humor in a well-controlled experimental setting, and (2) are realistic, musically “valid” melodies, conforming to heuristic Western melodic expectancies. But all things considered, although we tried to lower listeners’ time devoted to the experiment as much as possible, listening to 50 melody versions can still be tedious, and fatigue or boredom can counteract humor perception. Furthermore, there might always be a slight risk that self-report judgments on bipolar rating scales cannot provide strong objective results, despite that it has been shown that subjective, self-reported humor ratings positively correlate with psychophysical measures [67], [68].

In addition, we cannot completely rule out the possibility that the observed effects partly resulted from the perception of “global” variables of the melodies rather than the “local” variables of a phrase-ending schema. Global variables can be interpreted as latent variables: sum and direction of all steps in the melody, the key, the timbre, etc., that cannot be controlled within the experimental design. And indeed, as we already mentioned, the instructions of the experiment asked participants to “judge the whole melodies depending on the final note”. In other words, the melody as a whole might have contributed much more to listeners’ judgments than the closing note itself, and this might have influenced aesthetic category ratings to some extent (but not goodness of fit). However, if *only* the melodies would have affected the judgments and the final note would have been indifferent, then we would have experienced more uniform distribution among the ratings – but we could observe clear differences and tendencies instead. Furthermore, the rhythmic and intervallic differences between the two melodies, as well as the leading tone before the final note in the minor melody, might also have affected listeners’ humor ratings. As we mentioned in the Methods section, we intentionally wanted to create variability between the two melodies to avoid boredom effects, and the composed melodies still meet the requirements of Western listeners’ heuristic melodic expectancies (cf. [3 pp. 93–94]. To address the emerging questions presented above, namely whether a “playful mode” (such as in our case the major melody’s playful character) is required to perceive incongruencies as humorous and the results obtained were not affected by the differences of the two melodies, we created a follow-up experiment In this second experiment [69], we used the same melodies and experimental paradigm as in the current study, but transposed the original major melody to minor mode, and the minor melody to major mode. Thus, eliminated the global, latent variables without changing the melody, or the timbre. The preliminary results clearly corroborate our findings discussed above: the now major melody (originally: minor) had some positive humor ratings, while the new minor melody (originally: major) had only negative humor ratings (i.e., it was rated as “non-humorous”). The data is available at an OSF repository: https://osf.io/ujv28/?view_only=5c1fd3f2b4b74531a507ff0ec5228b15.

For further research and confirmation, another solution would be to use several different major and minor melodies with a smaller set of final-note changes. Because of the complexity of both music and humor, further experiments should also take into consideration further aspects of music such as rhythm or harmony. Also, we need to emphasize that our results are not universal but limited by the participants’ enculturation, in our case, Western musical culture.

## Conclusion

Humor is a very complex phenomenon with large subjective differences in its perception. In our experiment, we found that it is not the final-step related magnitude of change, or its incongruency level compared to the original final note, that mainly influences participants’ judgments: they also require a “playful context” to find it more humorous. The original melody that listeners found playful (i.e., the major melody) were found more humorous; as a result, the means of minor-mode humor ratings were almost exclusively at the negative, “not humorous” end of the bipolar scales. We also showed that participants found the final notes that finished lower than the original, “correct” tonic final note significantly less fitting and less humorous. Musicians’ and non-musicians’ had similar humor ratings, except for a few final notes that musicians found more humorous, most likely because of their more extended experience with music, which allows for building broader cognitive contextual frameworks that make it possible to cognitively resolve more incongruent final notes, leading to the perception of humor.

## Electronic supplementary material

Below is the link to the electronic supplementary material.

Supplementary Material 1

## Data Availability

The dataset supporting the conclusions of this article is available in the OSF repository: https://osf.io/3cxf4/?view_only=2daabc081f594bcd862558b0f38b43c2.

## Footnotes

1 This gag with the same song has been reused several times in animated series from Looney Tunes to South Park.

2 Perhaps the most famous historical example is Mozart’s “Ein musikalischer Spass” (K. 522), which clearly demonstrates the strong relationship between incongruency and musical humour on various semantic layers of the composition.

3 We need to highlight that a study like ours is naturally limited in scope. Among others, we would like to remind the reader of the fact that music is almost never a purely “auditory” phenomenon. Besides the often multisensory phenomenological experience of music perception, neuroscientific methods provide the evidence that even simple sounds can involve visual cortical areas and auditory areas can also be activated without the presence of a sound. These phenomena emphasize the importance of musical imagery in musical practice as well (for an important review see [70].

4 Note that throughout this article, we employ the closest possible English translation for each term used in the experiment.
