# Supplementary material for: Final-note expectancy and humor: an empirical investigation
Source: BMC Psychol. 2022 Sep 30;10:228. doi: 10.1186/s40359-022-00936-z (PMC9526306; doi:10.1186/s40359-022-00936-z)
Supplement: Supplementary file 1 — Supplementary Material 1 [file 40359_2022_936_MOESM1_ESM.docx]

**Supplementary material**

**Network analysis**

**Supplementary table 1**

*Summary of Network*

| Network | Number of nodes | Number of non-zero edges | Sparsity |
| --- | --- | --- | --- |
| *Musician–major* | 5 | 7/10 | 0.500 |
| *Non-musician–major* | 5 | 7/10 | 0.300 |
| *Musician–minor* | 5 | 5/10 | 0.600 |
| *Non-musician–minor* | 5 | 4/10 | 0.300 |

**Supplementary figure 1**

*Centrality Plot*

*
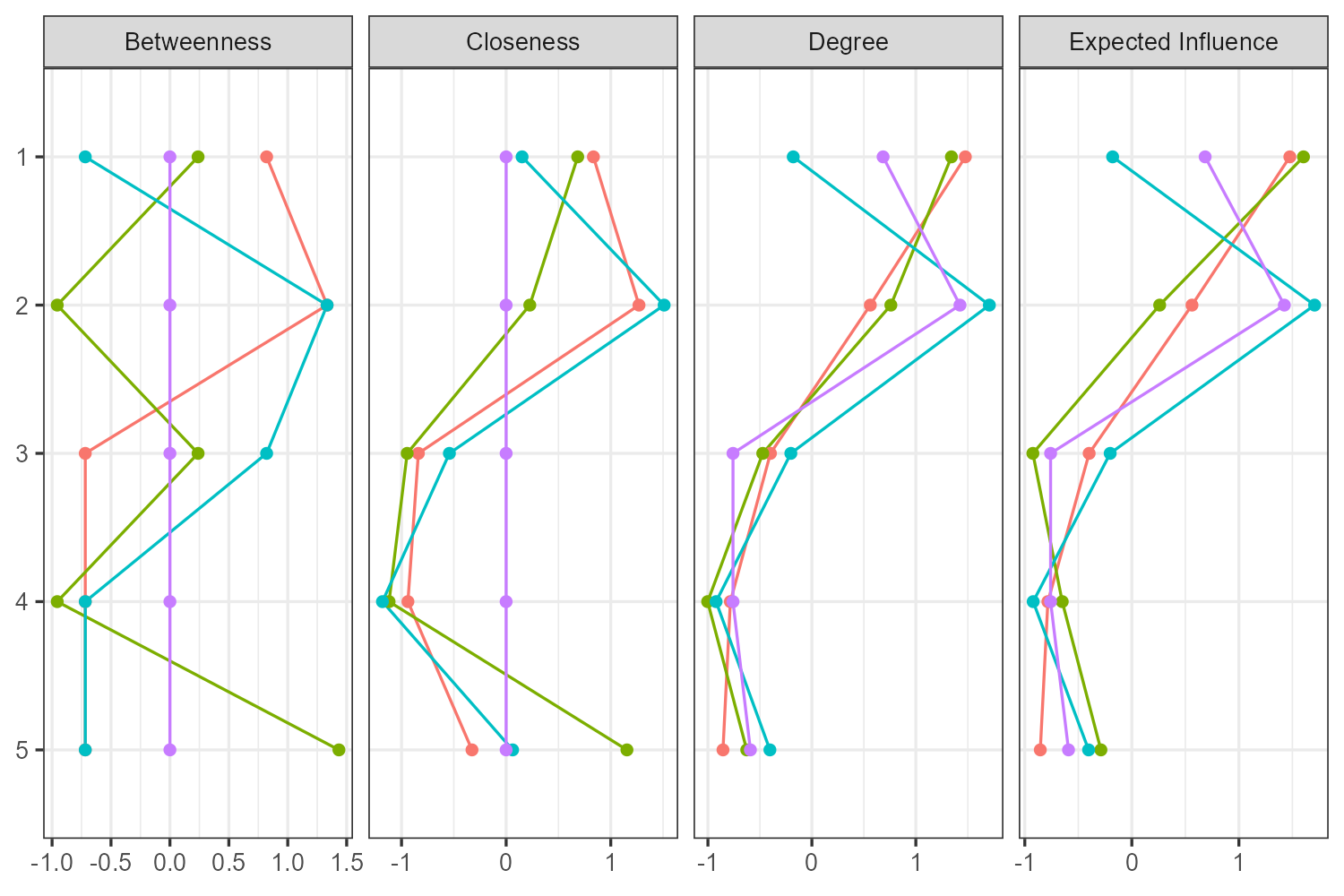
*

**Supplementary table 2**

*Centrality measures per variable*

| **Variable** | **Betweenness** | **Closeness** | **Strength** | **Expected influence** |
| --- | --- | --- | --- | --- |
|  | ***Non-musician–major*** | | | |
| goodness of fit | -0.718 | -0.326 | -0.855 | -0.855 |
| humor | -0.718 | -0.939 | -0.784 | -0.784 |
| playfulness | -0.718 | -0.839 | -0.399 | -0.399 |
| pleasantness | 1.334 | 1.270 | 0.562 | 0.562 |
| beauty | 0.821 | 0.834 | 1.476 | 1.476 |
|  | ***Non-musician–minor*** | | | |
| goodness of fit | 1.434 | 1.155 | -0.627 | -0.289 |
| humor | -0.956 | -1.119 | -1.003 | -0.650 |
| playfulness | 0.239 | -0.946 | -0.473 | -0.925 |
| pleasantness | -0.956 | 0.226 | 0.760 | 0.260 |
| beauty | 0.239 | 0.684 | 1.342 | 1.604 |
|  | ***Musician–major*** | | | |
| goodness of fit | -0.718 | 0.062 | -0.404 | -0.404 |
| humor | -0.718 | -1.182 | -0.922 | -0.922 |
| playfulness | 0.821 | -0.542 | -0.202 | -0.202 |
| pleasantness | 1.334 | 1.510 | 1.707 | 1.707 |
| beauty | -0.718 | 0.152 | -0.179 | -0.179 |
|  | ***Non-musician–major*** | | | |
| goodness of fit | 0.000 | 0.000 | -0.592 | -0.592 |
| humor | 0.000 | 0.000 | -0.759 | -0.759 |
| playfulness | 0.000 | 0.000 | -0.759 | -0.759 |
| pleasantness | 0.000 | 0.000 | 1.424 | 1.424 |
| beauty | 0.000 | 0.000 | 0.685 | 0.685 |

**Cluster Analysis**

***Non-musician – major mode***

**Supplementary table 3**

*Fuzzy C-Means Clustering*

| Clusters | N | R^2^ | AIC | BIC | Silhoette |
| --- | --- | --- | --- | --- | --- |
| 2 | 25 | 0.685 | 35.420 | 42.730 | 0.560 |

*Note.* The model is optimized with respect to the BIC value.

**Supplementary table 4**

*Cluster information*

| Cluster | 1 | 2 |
| --- | --- | --- |
| Size | 16 | 9 |
| Explained proportion within-cluster heterogeneity | 0.466 | 0.534 |
| Within sum of squares | 10.904 | 12.513 |
| Centroid Goodness of fit | -0.551 | 0.994 |
| Centroid Humor | -0.674 | 1.122 |
| Centroid Playfulness | -0.659 | 1.138 |

**Supplementary table 5**

*Cluster Means*

|  | Goodness of fit | Humor | Playfulness |
| --- | --- | --- | --- |
| Cluster 1 | -0.528 | -0.634 | -0.642 |
| Cluster 2 | 0.938 | 1.128 | 1.142 |

***Musician – Major mode***

**Supplementary table 6**

*Fuzzy C-means clustering*

| Clusters | N | R² | AIC | BIC | Silhouette |
| --- | --- | --- | --- | --- | --- |
| 2 | 25 | 0.656 | 38.720 | 46.030 | 0.520 |

*Note.*  The model is optimized with respect to the *BIC* value.

**Supplementary table 7**

*Cluster information*

| Cluster | 1 | 2 |
| --- | --- | --- |
| Size | 17 | 8 |
| Explained proportion within-cluster heterogeneity | 0.581 | 0.419 |
| Within sum of squares | 15.518 | 11.198 |
| Centroid Goodness of fit | -0.548 | 1.220 |
| Centroid Humor | -0.553 | 1.177 |
| Centroid Playfulness | -0.538 | 1.259 |

**Supplementary table 8**

*Cluster Means*

|  | Goodness of fit | Humor | Playfulness |
| --- | --- | --- | --- |
| Cluster 1 | -0.534 | -0.536 | -0.529 |
| Cluster 2 | 1.135 | 1.139 | 1.125 |

***Non-Musician – Minor***

**Supplementary table 9**

*Fuzzy C-means Clustering*

| Clusters | N | R² | AIC | BIC | Silhouette |
| --- | --- | --- | --- | --- | --- |
| 3 | 25 | 0.713 | 37.550 | 48.520 | 0.410 |

*Note.*  The model is optimized with respect to the *BIC* value.

**Supplementary table 10**

*Cluster Information*

| Cluster | 1 | 2 | 3 |
| --- | --- | --- | --- |
| Size | 4 | 7 | 14 |
| Explained proportion within-cluster heterogeneity | 0.194 | 0.138 | 0.668 |
| Within sum of squares | 3.783 | 2.700 | 13.067 |
| Centroid Goodness of fit | 1.377 | -0.955 | -0.051 |
| Centroid Humor | 1.291 | -1.254 | 0.196 |
| Centroid Playfulness | 1.246 | -1.191 | 0.174 |

**Supplementary table 11**

*Cluster Means*

|  | Goodness of fit | Humor | Playfulness |
| --- | --- | --- | --- |
| Cluster 1 | 1.600 | 1.279 | 1.287 |
| Cluster 2 | -0.965 | -1.256 | -1.183 |
| Cluster 3 | 0.025 | 0.263 | 0.224 |

**Musician -minor**

**Supplementary table 12**

*Fuzzy C-means Clustering*

| Clusters | N | R² | AIC | BIC | Silhouette |
| --- | --- | --- | --- | --- | --- |
| *3* | *25* | 0.548 | 46.260 | 57.230 | 0.360 |

*Note*.  The model is optimized with respect to the BIC value.

**Supplementary table 13**

*Cluster Information*

| Cluster | 1 | 2 | 3 |
| --- | --- | --- | --- |
| Size | 9 | 11 | 5 |
| Explained proportion within-cluster heterogeneity | 0.246 | 0.433 | 0.322 |
| Within sum of squares | 6.943 | 12.233 | 9.088 |
| Centroid Goodness of fit | -0.597 | -0.276 | 1.297 |
| Centroid Humor | 0.551 | -0.746 | 0.600 |
| Centroid Playful | 0.570 | -0.790 | 0.563 |

*Cluster Means*

|  | | Goodness of fit | | Humor | | Playful | |
| --- | --- | --- | --- | --- | --- | --- | --- |
| Cluster 1 |  | -0.653 |  | 0.494 |  | 0.563 |  |
| Cluster 2 |  | -0.111 |  | -0.823 |  | -0.884 |  |
| Cluster 3 |  | 1.420 |  | 0.920 |  | 0.931 |  |

**Supplementary figure 2**

*t-SNE plots of the clusters*

Non-musician – Major


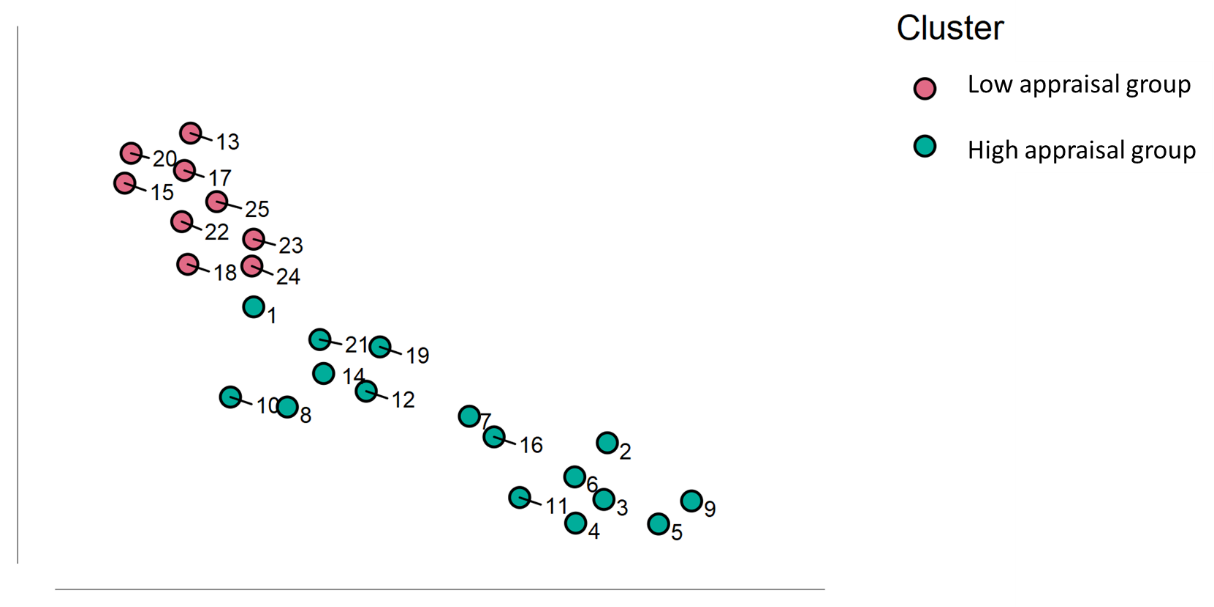


Musician – Major


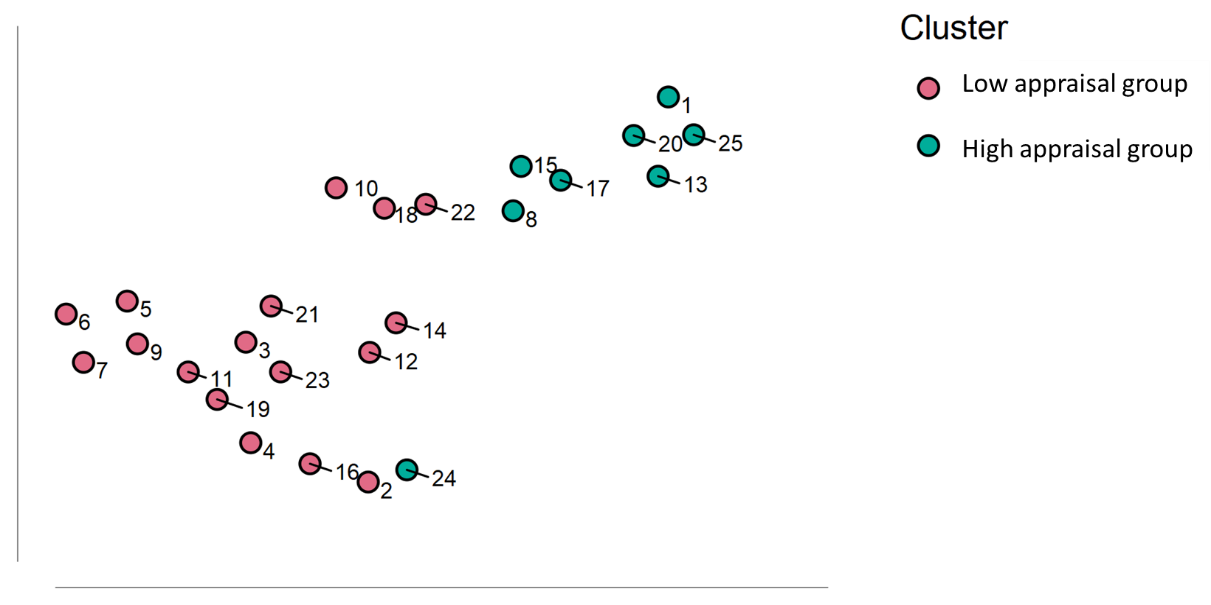


Plot numbers:

1: C_3_; 2: C#_3_; 3: D_3_; 4: D#_3_; 5: E_3_; 6: F_3_; 7: F#_3_; 8: G_3_; 9: G#_3_; 10: A_3_; 11: A#_3_; 12: B_3_; 13: C_4_; 14: C#_4_; 15: D_4_; 16: D#_4_; 17: E_4_; 18: F_4_; 19: F#_4_; 20: G_4_; 21: G#_4_; 22: A_4_; 23: A#_4_; 24: B_4_; 25: C_5_

Non-musician – Minor


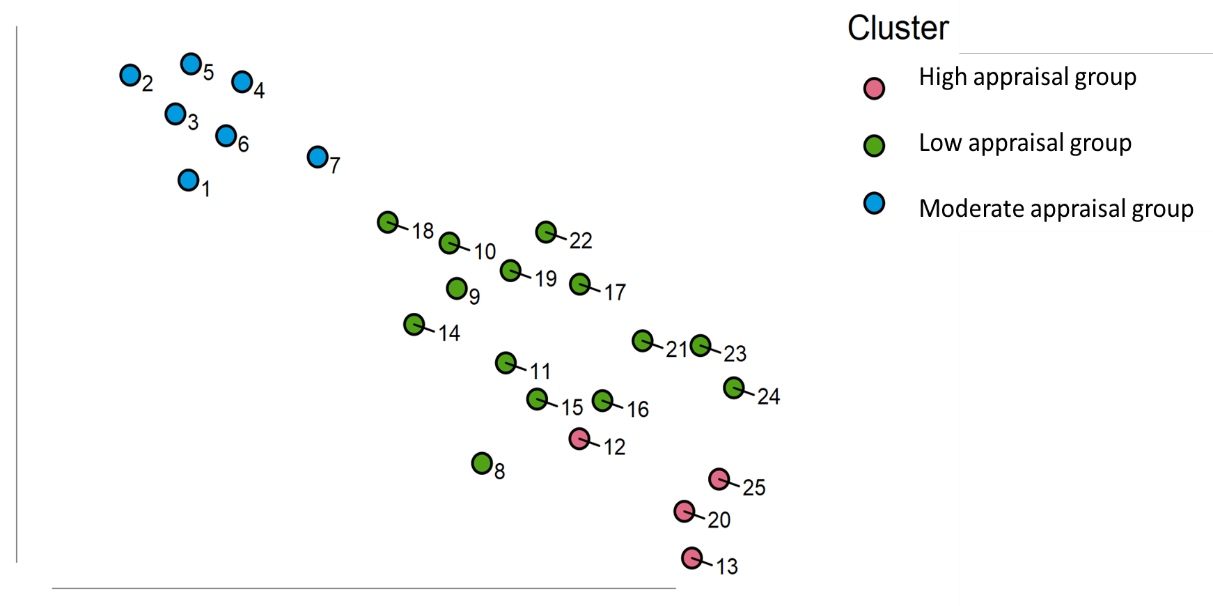


Musician – Minor


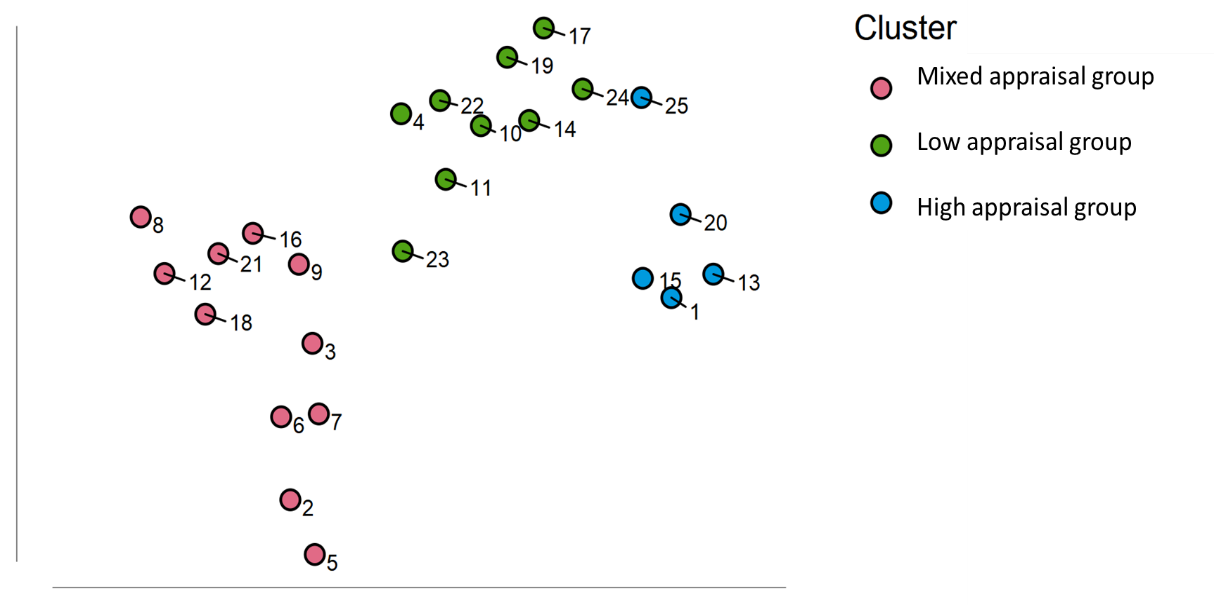


*Plot numbers:*

1: A_3_; 2: A#_3_; 3: B_3_; 4: C_4_; 5: C#_4_; 6: D_4_; 7: D#_4_; 8: E_4_; 9: F_4_; 10: F#_4_; 11: G_4_; 12: G#_4_; 13: A_4_; 14: A#_4_; 15: B_4_; 16: C_5_; 17: C#_5_; 18: D_5_; 19: D#_5_; 20: E_5_; 21: F_5_; 22: F#_5_; 23: G_5_; 24: G#_5_; 25: A_5_

*Note.* In t-SNE plots the X and Y axes are uninterpretable: t-SNE plots only seek to provide an impression of how high-dimesnional data can be mapped on two-dimensional space. It shows the relative distances between observations and clusters.

**Supplementary figure 3**

Color coded table

Major mode Minor mode

| **Final note** | **Musicians** | **Non-musicians** |  | **Final note** | **Musicians** | **Non-musicians** |
| --- | --- | --- | --- | --- | --- | --- |
| C_3_ |  |  |  | A_3_ |  |  |
| C#_3_ |  |  |  | A#_3_ |  |  |
| D_3_ |  |  |  | B_3_ |  |  |
| D#_3_ |  |  |  | C_4_ |  |  |
| E_3_ |  |  |  | C#_4_ |  |  |
| F_3_ |  |  |  | D_4_ |  |  |
| F#_3_ |  |  |  | D#_4_ |  |  |
| G_3_ |  |  |  | E_4_ |  |  |
| G#_3_ |  |  |  | F_4_ |  |  |
| A_3_ |  |  |  | F#_4_ |  |  |
| A#_3_ |  |  |  | G_4_ |  |  |
| B_3_ |  |  |  | G#_4_ |  |  |
| C_4_ |  |  |  | A_4_ |  |  |
| C#_4_ |  |  |  | A#_4_ |  |  |
| D_4_ |  |  |  | B_4_ |  |  |
| D#_4_ |  |  |  | C_5_ |  |  |
| E_4_ |  |  |  | C#_5_ |  |  |
| F_4_ |  |  |  | D_5_ |  |  |
| F#_4_ |  |  |  | D#_5_ |  |  |
| G_4_ |  |  |  | E_5_ |  |  |
| G#_4_ |  |  |  | F_5_ |  |  |
| A_4_ |  |  |  | F#_5_ |  |  |
| A#_4_ |  |  |  | G_5_ |  |  |
| B_4_ |  |  |  | G#_5_ |  |  |
| C_5_ |  |  |  | A_5_ |  |  |

*Note.* Blue: High Humor level, Playfulness level and Goodness of fit;

Red: Low Humor level, Playfulness level and Goodness of fit;

Green: High Humor and Playfulness levels but low Goodness of fit;

Yellow: Moderate Humor and Playfulness levels with moderate Goodness of fit ratings.

**Labels of the bipolar scales in Hungarian language**

| ***Hungarian terms*** | ***English translation*** |
| --- | --- |
| Egyáltalán nem illet bele – Teljesen beleillett | Did not fit at all – Absolutely fit into it |
| Csúnya – Szép | Ugly – Beautiful |
| Komoly – Játékos | Serious – Playful |
| Nem humoros – Humoros/Vicces | Not humorous – Humorous/Funny |
| Irritáló - Kellemes | Irritating – Pleasant |
